# A hybrid model of the primary visual cortex

**DOI:** 10.1186/1471-2202-12-S1-P184

**Published:** 2011-07-18

**Authors:** Martin Rehn, David Silverstein, Jan Olmårs, Anders Lansner

**Affiliations:** 1 Department of Computational Biology, Royal Institute of Technology, SE-114 21, Stockholm, Sweden; 2 Department of Computational Biology, Stockholm University, SE-114 21, Stockholm, Sweden

## 

As computer power grows, so can the scale of biophysical simulations of networks of neurons in the cerebral cortex. As cell physiology and microanatomy of the cortical circuitry are better understood, the realism of such models can be enhanced. Previously, we have demonstrated that experimental bottom-up information is not enough to specify a cortical network model that captures realistic network dynamics. As a complement, we suggested using top-down information based on functional hypotheses of brain function, or abstract neural network models [1]. Here we present such a *hybrid* bottom-up/top-down model for the mammalian primary visual cortex. We have implemented this model in NEURON and simulations are carried out on an IBM BG/L supercomputer.

**Figure 1:**
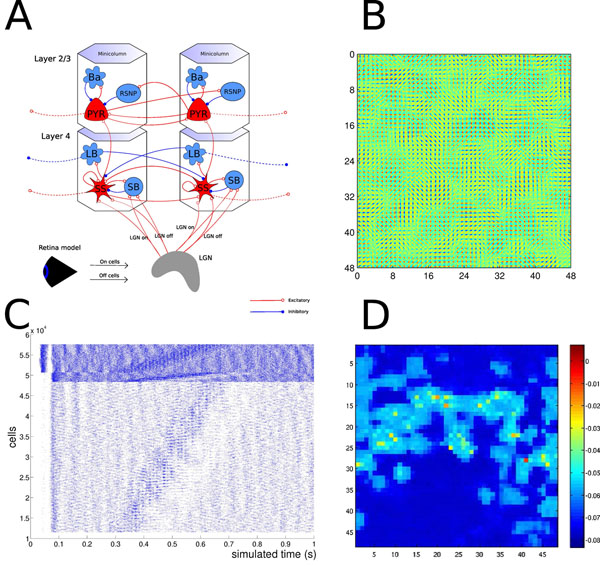
Model structure and simulation results. **A**. Overview of the model showing retina, LGN, and a cortical microcircuit with cell types. **B.** Orientation preference map for neurons in layer 4. **C.** Network response to a moving bar visual stimulus. **D.** Snapshot of a simulated “voltage sensitive dye” image of the activity in layer 2/3, in response to a stationary bar stimulus, illustrating a “virtual experiment” in the model.

Our model incorporates layer 4 and layer 2/3 sections of the cortical sheet. In addition, we use a multilayer retina model and a simple representation of the LGN [2]. See figure 1A. The cell models are of Hodgkin-Huxley type, with a small number of compartments, and several classes of ion channels. There are six cell types; spiny stellate cells, pyramidal cells, and four types of inhibitory interneurons [1]. While we model a cascade of cortical areas, our focus is on understanding computation in one area of the primary visual cortex.

On top of the biophysical model, we impose connectivity from the LISSOM model, which is a self-organizing map model, and from a generalized associative memory model [3,4]. We find that much of the computational properties of the abstract models carry over to the biophysical simulation (figure 1B-1C).
